# Level of Fatty Acid Binding Protein 5 (FABP5) Is Increased in Sputum of Allergic Asthmatics and Links to Airway Remodeling and Inflammation

**DOI:** 10.1371/journal.pone.0127003

**Published:** 2015-05-28

**Authors:** Hille Suojalehto, Pia Kinaret, Maritta Kilpeläinen, Elina Toskala, Niina Ahonen, Henrik Wolff, Harri Alenius, Anne Puustinen

**Affiliations:** 1 Occupational Medicine Team, Finnish Institute of Occupational Health, Helsinki, Finland; 2 Unit of Systems Toxicology, Finnish Institute of Occupational Health, Helsinki, Finland; 3 Department of Pulmonary Diseases and Allergology, University of Turku, Turku, Finland; 4 Department of Otolaryngology- Head and Neck Surgery, Temple University, Philadelphia, United States of America; University of Southampton School of Medicine, UNITED KINGDOM

## Abstract

**Background:**

The inflammatory processes in the upper and lower airways in allergic rhinitis and asthma are similar. Induced sputum and nasal lavage fluid provide a non-invasive way to examine proteins involved in airway inflammation in these conditions.

**Objectives:**

We conducted proteomic analyses of sputum and nasal lavage fluid samples to reveal differences in protein abundances and compositions between the asthma and rhinitis patients and to investigate potential underlying mechanisms.

**Methods:**

Induced sputum and nasal lavage fluid samples were collected from 172 subjects with 1) allergic rhinitis, 2) asthma combined with allergic rhinitis, 3) nonallergic rhinitis and 4) healthy controls. Proteome changes in 21 sputum samples were analysed with two-dimensional difference gel electrophoresis (2D-DIGE), and the found differentially regulated proteins identified with mass spectrometry. Immunological validation of identified proteins in the sputum and nasal lavage fluid samples was performed with Western blot and ELISA.

**Results:**

Altogether 31 different proteins were identified in the sputum proteome analysis, most of these were found also in the nasal lavage fluid. Fatty acid binding protein 5 (FABP5) was up-regulated in the sputum of asthmatics. Immunological validation in the whole study population confirmed the higher abundance levels of FABP5 in asthmatic subjects in both the sputum and nasal lavage fluid samples. In addition, the vascular endothelial growth factor (VEGF) level was increased in the nasal lavage fluid of asthmatics and there were positive correlations between FABP5 and VEGF levels (r=0.660, p<0.001) and concentrations of FABP5 and cysteinyl leukotriene (CysLT) (r=0.535, p<0.001) in the nasal lavage fluid.

**Conclusions:**

FABP5 may contribute to the airway remodeling and inflammation in asthma by fine-tuning the levels of CysLTs, which induce VEGF production.

## Introduction

Asthma and rhinitis are highly prevalent chronic inflammatory airway diseases that often coexist; most patients with asthma also suffer from rhinitis, and conversely many patients with allergic rhinitis (AR) also have nonspecific bronchial hyperresponsiveness [1]. Allergic asthma and AR share several common mediators which contribute to both upper and lower airway inflammation [2]. For example, the immunoglobulin E (IgE)- dependent activation of mast cells; the infiltration of eosinophils; the increase in the numbers of Cd4+ lymphocytes and Th2 type cytokine concentrations as well as in the cytokines associated to regulatory T cells, Th1 and Th17 cells, are common characteristics in both conditions [3].

Induced sputum (IS) contains a cellular part and a fluid phase; it is a non-invasive, standardized, validated method of sampling airway cells and secretions [4–6]. Sputum eosinophilia is a characteristic feature of asthma and can also be detected in AR, whereas sputum neutrophilia is detected in moderate severe asthma and in chronic airway obstruction in asthma [7–9]. The fluid phase of sputum has been less well characterized. It includes proteins, many of which have been linked to inflammatory airway diseases [10]. Several inflammatory markers, including eosinophil cationic protein, interleukins 8 and 13, and cysteinyl leukotrienes (CysLTs), have been detected in the fluid phase of sputum collected from asthmatics.

Proteomic technologies can be considered complementary to the genomic approaches in the identification of the molecular signatures associated with clinically important disease phenotypes. Proteomic studies of human biofluids could lead to the identification of novel biomarkers while measuring the presence and abundance of the functionally-relevant proteins associated with the disease. The epithelial surface of the airways is one of the crucial physico-chemical and immunological barriers in the body that respond to the external atmosphere, thus the respiratory system is inevitably an ideal location for exploring pulmonary diseases and their complications. A few studies have applied non-invasive methods such as IS and nasal lavage fluid (NLF) to characterize proteomic changes in upper airway diseases [11–15]. It is known that these specimens contain proteins secreted from epithelial and inflammatory cells, and therefore their analysis in the context of asthma may reveal important biological activities related to airway hyperresponsiveness, remodeling and inflammation.

The present study assessed the IS proteome from patients with asthma and AR and compared it to the IS collected from AR or nonallergic rhinitis (NAR) patients without concomitant asthma, as well as to that from healthy subjects. Our aim was to determine whether the proteomics method could reveal different protein expressions in the disease groups and thus reveal potential candidate biomarkers. We hypothesized that such markers could be found in both the upper and lower airways in asthma and rhinitis in accordance with the “unified airway” concept and we further validated the candidate biomarkers in both IS and NLF.

## Materials and Methods

### Study design

**Fig 1:**
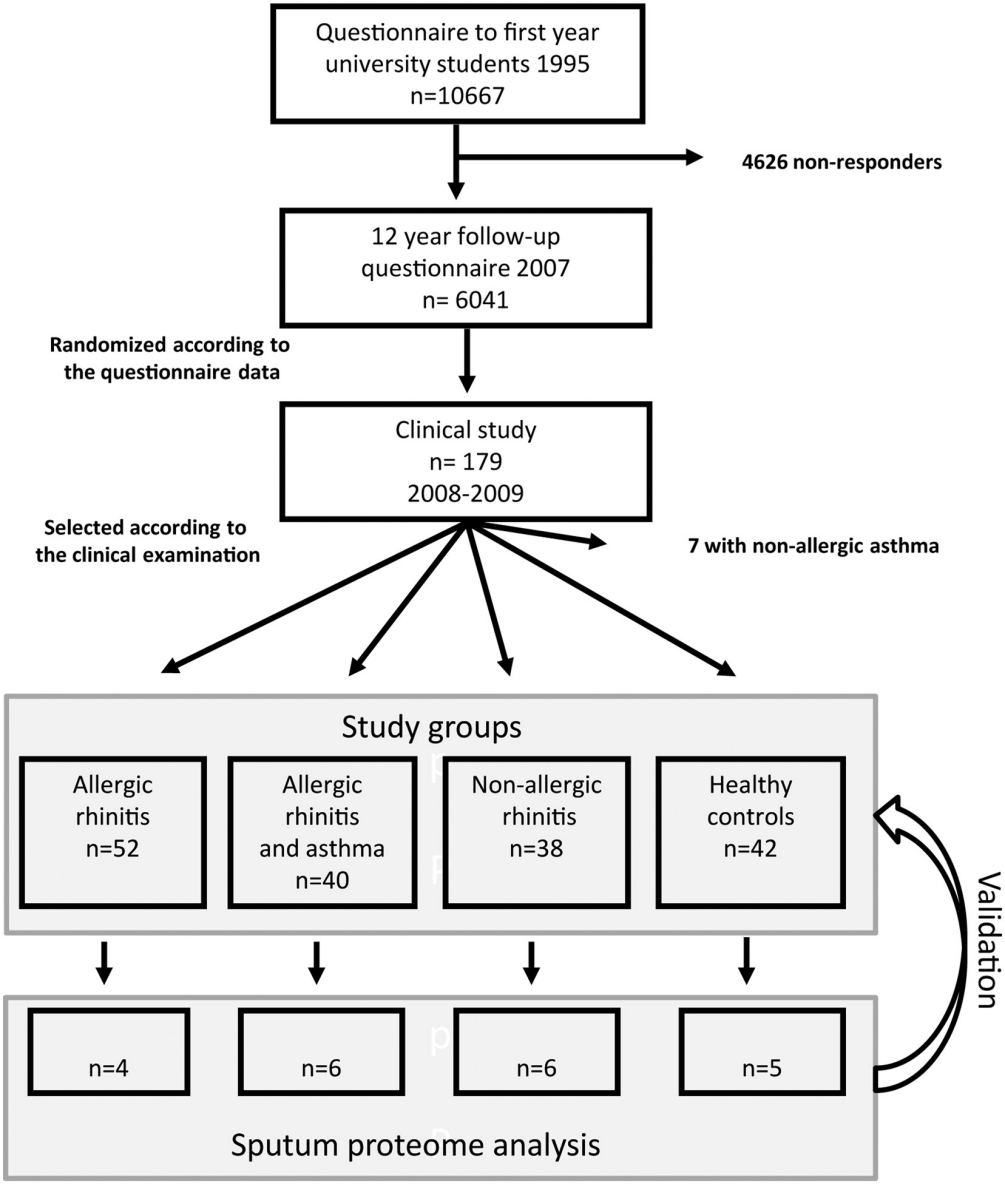
Study flow chart.

The study population was selected from a cohort of first-year university students followed up for 12 years, and described in detail in a recent article (Fig 1)[16]. The study groups were: 1) AR 2) AR combined with asthma (AR+asthma) 3) NAR and 4) healthy controls. The inclusion criteria for the AR group were at least one positive (≥ 3mm) skin prick test (SPT) and relevant rhinitis symptoms caused by the allergen. In the case that the wheal in the negative control was ≥2 mm, sensitization was confirmed with the specific IgE. The AR+asthma group participants fulfilled the AR group criteria and had previous doctor-diagnosed asthma or ≥12% reversibility in forced expiratory flow in one second (FEV1) after bronchodilator administration in combination with asthma symptoms. The NAR group consisted of subjects with periodic or perennial rhinitis symptoms not related to allergens, asthmatics were excluded. The subjects in the control group had no diagnosed chronic respiratory diseases and no recurrent or persistent respiratory symptoms. We selected samples from 4–6 nonsmoking participants from each group for proteomic analysis of sputum (< 60 squamous cells / 200 nonsquamous cells) and NLF (Fig 1). All collected specimens were included in the immunological validation.

The study was approved by the ethical committee of the Turku University and Turku University Central Hospital (19/180/2008). All participants provided written informed consent.

### Clinical examination

Participants filled in a self-completed questionnaire on their medical history, respiratory and nasal symptoms, asthma severity, medication and smoking. A chest physician and rhinologist interviewed and examined them all. Short-acting β-agonists were withheld for 12 hours, long-acting β-agonists for 48 hours, leukotriene antagonists for 3 days and antihistamines for 7 days before the examination. Nasal and inhaled steroids were withheld for two weeks if possible (three subjects had shorter cessation of nasal steroids and five had shorter cessation of inhaled steroids).

### Skin prick tests and IgE measurements

The SPT panel included a negative control, a positive control (histamine) and standardized antigens of birch, alder, timothy grass and mugwort pollen, *Alternaria Alternata*, *Aspergillus fumigatus*, cat, dog and horse epithelium and house-dust mite *Dermatophagoides pteronyssimus* (ALK, Abello, Nieuwegein, The Netherland). Results were regarded as positive if the mean wheal diameter was at least 3 mm and the negative control wheal was < 2 mm. Serum total and specific IgEs were measured using the Phadia UniCAP System (Phadia, Uppsala, Sweden). A specific IgE of < 0.35 kU/l was regarded as normal.

### Lung function and exhaled nitric oxide

Flow-volume spirometry and a bronchodilation test were performed according to the guidelines [17], using a standard spirometer (Spirostar USB Medikro, Finland). The predicted values assessed for Finnish population were used. We measured exhaled nitric oxide was measured using an on-line chemiluminescence analyser (NIOX, Aerocrine AB, Solna, Sweden) in accordance with ATS/ERS recommendations [18].

### Induced sputum

Sputum was induced with hypertonic saline according to the recommended guidelines [5]. After collecting the sputum samples, a smear sample was taken for cell count and type analysis. The remaining samples were immediately preprocessed by isolating the cells from the mucus. Dithiothreitol (DTT) (Sputolysin reagent, KGaA /Calbiochem, Darmstadt, Germany) and water were added to facilitate the handling and processing of the sample. The samples were incubated for 45 minutes and pre-filtered through nylon cloth after which they were centrifuged (500g x) to remove cells. The supernatant was filtered through 0.45 μm (Millex-hv PVDF, Millipore) and frozen to -80°C for further use. As DTT disturbs colorimetric protein concentration measurements, protein concentration was approximated from SDS-PAGE gel; the intensities of IS samples (Image Quant 1D TL 7.0 software, GE Healthcare, Uppsala, Sweden) to known amounts of molecular weight markers were compared to obtain the proportional concentration of the samples, which varied between 0.5 and 1 mg/ml. Similar IS protein amounts indicated no differences in cell lysis between the groups. Samples were concentrated five-fold using ultracentrifugal concentrator tubes (VivaSpin 4, 5000 MWCO PES, Sartorius Stedim Biotech, Goettingen, Germany). A total of 100 μg of each concentrated sample was used for proteomic analysis and 3 μg of the untreated sputum for immunological validation with Western blotting.

### Nasal lavage fluid

We used 7.5 ml of saline for washing each nasal cavity, which totalled 15 ml of saline per person. The collected fluid samples were combined and centrifuged at 500g x to separate the cells for cell counting and cell type analysis. The supernatant was centrifuged for the second time at 4000g x, before it was filtered through the 0.45 μm membrane (Millex-hv PVDF, Millipore) and divided into aliquots. Samples were frozen to -70°C for further use. Protein concentrations (RC DC Protein Assay, BioRad) of the NFL samples varied between 0.03 and 0.19 mg/ml. A total of 7–9 ml of each NLF sample was concentrated with ultrafiltration (Amicon Ultra-15 5000 MWCO, Millipore, Ireland) to 250 μl which was used in the proteomic analysis. For immunoblotting, 12 μl of untreated NFL was loaded onto the gel lane and for ELISA 250 μl was evaporated in SpeedVac to 50 μl.

### Two-dimensional differential gel electrophoresis (2D-DIGE) and expression analysis

The protein samples were cleaned of salts, detergents and lipids by the ReadyPrep 2-D Cleanup kit (Bio-Rad Laboratories Inc. Hercules, CA) [19]. After purification, the protein samples were suspended to 2 μg/μL DIGE labeling buffer (30mM Tris, 7M Urea, 2M Thiourea, 4% 3-[(3-cholamidopropyl)dimethylammonio]-1-propanesulfonate (CHAPS)) and individual participant samples were randomly labeled with CyDye DIGE Fluor minimal dyes Cy3 and Cy5 (GE Healthcare) [19]. We used Cy2 dye for the pooled internal standard, which contained equal amounts of protein from each subject’s sample. The labelled samples were absorbed into 18 cm-long Immobiline DryStrips pH3-10 (GE Healthcare) using the cup loading method after rehydrating the strips for six hours with rehydration buffer (7M Urea, 2M Thiourea, 4% CHAPS, 0.04% bromophenol blue, 2% DTT, 2% immobilized pH gradient (IPG) buffer) [20, 21]. Proteins were separated vertically according to their isoelectric points with ETTAN IPGphor II (Amersham Biosciences, Uppsala, Sweden), starting with a direct current of 150 volts and increasing the voltage in five steps over 19 hours to 8000 volts yielding the total run time of 39000 Volt-hours. Prior to SDS-PAGE, the strips were equilibrated for 15 min in 50mM Tris pH 8.8, 6M urea, 30% glycerol, 2% SDS, 0.004% bromophenol blue, 1% DTT on a rocking table and for 15 min in the same solution containing 2% iodoacetamide (GE Healthcare) instead of DTT. SDS-PAGE runs of the strips were performed with ETTAN DALT (Amersham Biosciences) using 15 Watts per gel. The protein gels were scanned with an Ettan DIGE imager (GE Healthcare) immediately after gel electrophoresis at a resolution of 40 μm, and by applying specific excitation/emission wavelengths to the dyes: 540/595 nm for Cy3, 635/680 nm for Cy5 and 480/530 nm for Cy2. Spot detection, quantification and the search for abundance differences were conducted using DeCyder 2-D Differential Analysis Software v.7.0 (GE Healthcare).

### Gel Spot Protein Identification by Tandem Mass Spectrometry

After silver staining the gels, protein spots with ≥ |1.5| fold change in abundance and with Student’s *t*-test *p*-value ≤ 0.05 between different study group comparisons were selected for identification by mass spectrometry. In-gel trypsin digested peptides were extracted from gel pieces and analysed using liquid chromatography tandem mass spectrometry (LC—MS/MS) (nanoLC coupled to quadrupole-time-of flight (Q-TOF), Waters) in accordance with Rostila et al. [22]. We applied the Mascot program (Matrix Science Ltd) with a SwissProt database to identify the proteins from the LC-MS/MS files.

### Immunological validation

Fatty acid binding protein 5 (FABP5) abundance was validated by western blotting for 138 IS and 158 NLF samples. Western blotting was performed as described in Rostila et al. using precast 26-well 12% Criterion TGX gels (BioRad) [22]. Anti-FABP5 (Abcam #ab84028) primary antibody dilution was 1:800. Immunoblots were stained with anti-rabbit peroxidase-conjugated immunoglobulin (1:1000) (Dako Cytomaton) and chemiluminescent HRP-substrate ECL detection reagent (Perkin Elmer). They were visualized byan Image Quant LAS 4000 mini quantitative imager (GE Healthcare Biosciences). ImageQuant TL (GE Healthcare Biosciences) was used to analyse the intensities of the protein bands. We performed vascular endothelial growth factor (VEGF) and CysLT ELISA assays (Invitrogen) in accordance with the manufacturer´s instructions, using five-fold concentrated NLF in the analysis of 90 samples.

### Statistical analysis

Continuous variables were expressed as means (±standard deviation) and categorical values as percentages. The differences between the groups were analysed using the Kruskal-Wallis, Anova, the Chi-square test, Student´s t-test or the Mann-Whitney U-test, depending on their distribution. Bonferroni correction was used for multiple comparisons. We computed Pearson or Spearman's correlation between continuous variables. Receiver operating characteristic (ROC) curve was used to express the sensitivity and the specifity of FABP5 in predicting asthma. A p-value of < 0.05 was considered statistically significant. IBM SPSS Statistics for Windows, Version 20.0 (Armonk, NY: IBM Corp.) software was used for analysing the clinical parameters and for correlations. Hierarchical clustering of the identified differentially abundant proteins was performed with DeCyder-software’s Extended Data Analysis (EDA), 2-D Differential Analysis Software v.7.0 (GE Healthcare) using average linkage and the Euclidean metric as a distance measure. Statistical analyses for Western blot and ELISA were performed using GraphPad Prism 5 software (GraphPad Software). Intensity values from Western blot validation (FABP5 in IS and in NLF) and concentrations from ELISA (CysLT and VEGF in NLF) were imported to R software version 3.0.2, and after quantile normalization, principal component analysis (PCA) using the Bioconductor package limma [23] was applied to represent the relations (Euclidean distances) between the study groups.

## Results

### Study population

A total of 172 subjects enrolled in the study (Fig 1), the characteristics of whom are presented in Table 1. The mean age of the participants was 33 years; 64% of them were women. Most of the subjects in the AR and asthma with AR groups were sensitized to perennial allergens. No significant differences in smoking habits or spirometric values were detected between the groups. The exhaled nitric oxide level was significantly higher in the asthmatic group as compared to the NAR group (p = 0.011). Twenty-five asthma patients (62.5%) had not used any asthma medication or only a short-acting bronchodilator during the previous month, 14 asthmatics (35.0%) had used inhaled steroids during the time prior to the request to stop taking their medications.

**Table 1:** Characteristics of study subjects.

	Total (n = 172)	Allergic rhinitis (n = 52)	Asthma and allergic rhinitis (n = 40)	Nonallergic rhinitis (n = 38)	Control (n = 42)	P-value
Women, n (%)	110 (64.0)	35.(67.3)	19 (47.5)	31 (81.6)	25 (59.5)	0.015
Age (year), mean (SD)	33.1 (1.5)	33.2 (1.4)	32.5 (1.0)	33.1 (1.3)	33.5 (1.8)	0.023
Body-mass index (kg/m^2^), mean (SD)	24.0 (3.8)	24.3 (4.4)	24.2 (2.8)	23.5 (3.6)	23.8 (3.9)	0.751
Current smokers, n (%)	14 (8.1)	4 (7.7)	2 (5.0)	3 (7.9)	5 (11.9)	0.720
Number of positive SPTs						<0.001
1–3 positive SPTs, n*(%)	41 (24.7)	20 (40.0)	12 (30.8)	7 (19.4)	2 (4.9)	
At least 4 positive SPTs, n* (%)	57 (34.3)	30 (60.0)	27 (69.2)	0 (0.0)	0 (0.0)	
At least one positive SPT to perennial allergens, n* (%)	74 (44.6)	35 (70.0)	35 (89.7)	2 (5.6)	2 (4.9)	<0.001
Total IgE (kU/L), mean (SD)	125.3 (265.8)	209.8 (409.0)	199.5 (230.1)	35.6 (64.3)	31.2 (39.6)	<0.001
FVC% predicted, mean (SD)	97.7 (10.9)	96.1 (10.2)	101.0 (10.8)	96.4 (11.6)	97.5 (10.6)	0.146
FEV1% predicted, mean (SD)	92.8 (10.1)	92.8 (9.3)	94.3 (10.2)	90.8 (10.6)	93.3 (10.4)	0.473
FEV1/FVC, mean (SD)	79.8 (5.9)	81.0 (5.2)	78.2 (6.3)	79.4 (5.8)	80.1 (6.3)	0.151
FeNO (ppb), mean (SD)	16.8 (13.0)	17.4 (10.9)	22.1 (14.6)	12.8 (5.6)	14.7 (16.6)	0.011

*Patients with a negative control wheal of ≥ 2mm were excluded from skin prick test (SPT) analysis n = 166. IgE, immunoglobulin E; FEV1, forced expiratory volume in one second; FVC, forced vital capacity, FeNO, exhaled nitric oxide

All the 21 subjects selected for sputum proteomic analysis were non-smokers. Most subjects in the AR and AR+asthma groups were sensitized to multiple allergens, including perennial allergens (Table 2). The mean spirometric values and exhaled nitric oxide level were within normal limits in all groups. Six asthmatics (85.7%) had used only a short-acting bronchodilator or no asthma medication during the past month. The number of sputum eosinophils differed between the groups (p = 0.015): AR (mean 2.4, SD 2.9), asthma (mean 3.1, SD 5.3), NAR (mean 0.1, SD 0.1), and control (mean 0.3, SD 0.4). The number of eosinophils was significantly higher in the asthmatic group than that in the NAR group (p = 0.002) and to the control group (0.048). We detected no statistically significant difference in the numbers of sputum neutrophils (p = 0.846) or total cell count (p = 0.863) between the groups.

**Table 2:** Characteristics of subjects selected for proteomic analysis.

	Allergic rhinitis (n = 4)	Asthma and allergic rhinitis (n = 6)	Nonallergic rhinitis (n = 6)	Control (n = 5)	P-value
Women, n (%)	3 (75.0)	5 (83.3)	6 (100.0)	3 (60.0)	0.233
Age (year) mean (SD)	32.3 (1.3)	32.8 (1.2)	32.8 (1.2)	33.2 (1.9)	0.793
Body-mass index (kg/m^2^), mean (SD)	25.1 (6.0)	23.2 (2.6)	23.0 (3.4)	24.7 (3.8)	0.768
Number of positive SPTs					<0.001
1–3 positive SPTs, n (%)	1 (25.0)	2 (33.3)	0 (0.0)	0 (0.0)	
At least 4 positive SPTs, n (%)	3 (75.0)	4 (66.7)	0 (0.0)	0 (0.0)	
At least one positive SPT to perennial allergens, n (%)	3 (75.0)	5 (83.3)	0 (0.0)	0 (0.0)	0.005
Total IgE (kU/L) mean (SD)	754.5* (1308.0)	108.8 (101.3)	8.3 (5.0)	25.8 (32.4)	0.011
FVC% predicted, mean (SD)	97.8 (6.2)	103.0 (11.0)	98.7 (4.5)	96.2 (9.8)	0.585
FEV1% predicted, mean (SD)	89.0 (8.8)	95.2 (8.7)	95.0 (5.2)	94.0 (12.8)	0.721
FEV1/FVC, mean (SD)	76.6 (10.2)	78.1 (7.5)	81.7 (3.3)	81.6 (6.4)	0.581
FeNO (ppb), mean (SD)	15.1 (7.8)	22.1 (9.8)	9.9 (1.7)	11.7 (5.1)	0.032

* One subject had concurrent atopic exzema and total IgE 2713 kU/l: Total IgE varied between 30 and 210 kU/l in the other subjects in the allergic rhinitis group. SPT, skin prick test; IgE, immunoglobulin E; FVC, forced vital capacity; FEV1, forced expiratory volume in one second; FeNO, exhaled nitric oxide

### Two-dimensional differential gel electrophoresis (2D-DIGE) changes in protein abundances between study groups

**Fig 2:**
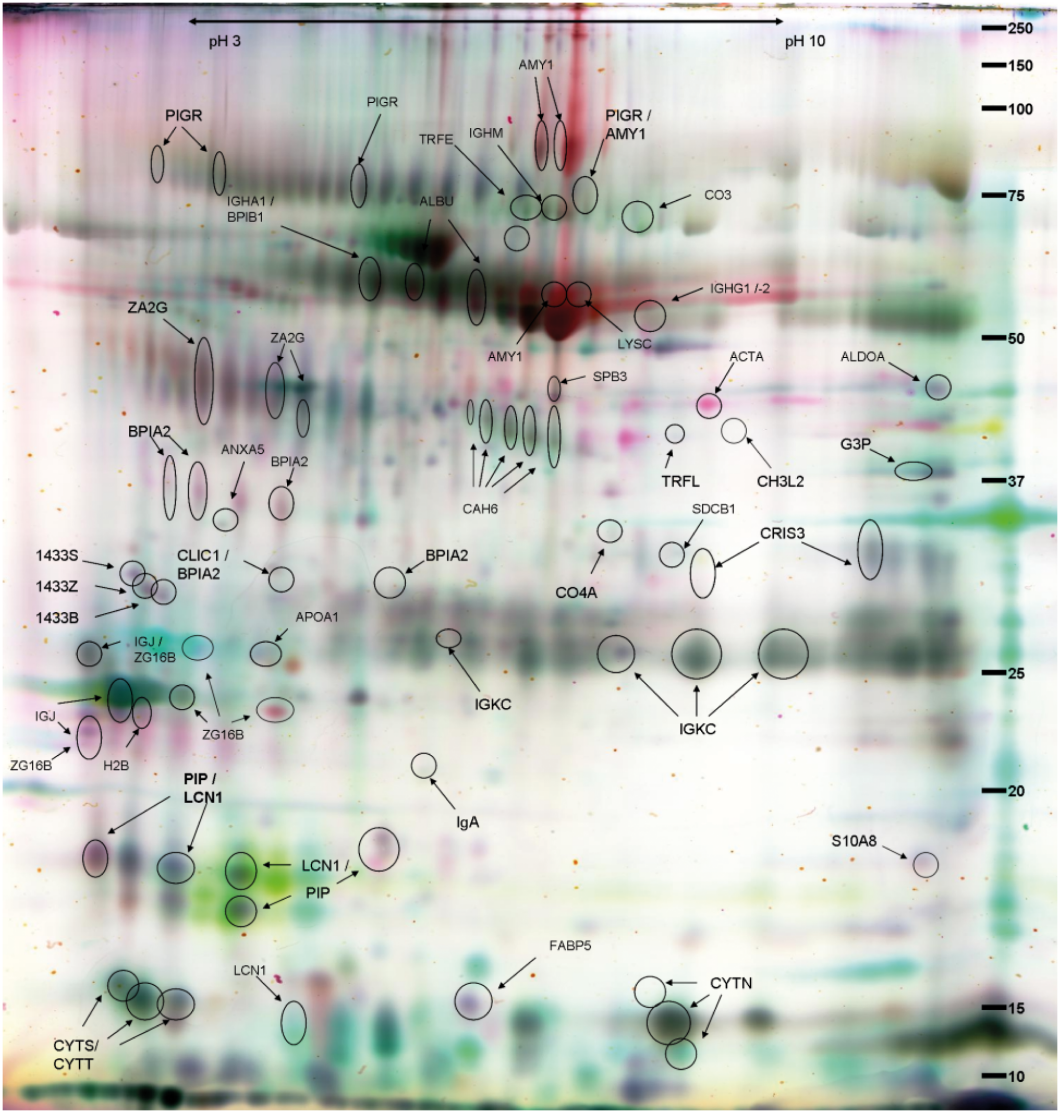
False colour two-dimensional differential gel electrophoresis (2D-DIGE) image of identified protein spots in sputum. False colour image of the identified protein spots (circled and named) on a 2-D DIGE-gel of the sputum fluid phase samples of 21 subjects subdivided as follows: asthma with allergic rhinitis, allergic rhinitis, nonallergic rhinitis, and healthy controls. Samples of the subjects with asthma with allergic rhinitis (reddish up-regulated) and with nonallergic rhinitis (green up-regulated), as well as the internal standard are shown. The identified proteins are listed in S1 Table.

Altogether ~1000 spots for IS and ~1400 spots for NLF proteins were matched between the scanned 2D-gels. By analysing the protein spots from the scanned gels, we discovered more than 80 up- or downregulated protein spots (fold change ≥|1.5|, Student’s *t*-test *p* < 0.05) for IS (Table 3 and S1 Table), and similarly 63 for NLF when compared against the internal standard. After LC-MS/MS analysis and Mascot search, a total of 31 different proteins were identified from the IS gels (Fig 2). All the proteins identified from the NLF gels were also found in the IS samples. Among the fifteen sputum specific proteins were several saliva proteins.

**Table 3:** Relationships between differently abundant sputum proteins in study groups.

Protein Name	UniProt AC	Asthma+AR vs. Control Av. Ratio	AR vs. Control Av. Ratio	NAR vs. Control Av. Ratio	Asthma+AR vs. AR Av. Ratio	Asthma+AR vs. NAR Av. Ratio
Zymogen granule protein 16 homolog B	Q96DA0	2.59			3.18	
Protein S100-A8	P05109	1.82			1.74	-1.7
Ig gamma-1 chain C region	P01857	-1.5				
Lipocalin-1	P31025	-1.61	-2.1			
Serum albumin	P02768	-1.66	2.01		-3.35	
Complement C4-A	P0C0L4	-1.78	-1.58			-1.8
Complement C3	P01024	-2.03				
Chitinase-3-like protein 2	Q15782	-2.35				-2.48
Ig alpha-1 chain C region	P01876	-2.41				
Fructose-bisphosphate aldolase A	P04075	-2.54			-3.05	
Alpha-amylase	P04746	-3.42	-3.41			
Carbonic anhydrase 6	P23280	-5.31	-5.1			
BPI fold-containing family B member 1	Q8TDL5		1.88		-2.74	
Fatty acid-binding protein, epidermal	Q01469		-1.55		2.15	
Annexin A5	P08758		-1.89			
Prolactin-inducible protein	P12273		-1.9		1.74	
Cysteine-rich secretory protein 3	P54108		-1.91			
Trypsin-3	P35030		-1.91			
Serpin B4	P48594		-1.98			
Syntenin-1	O00560		-2.35		1.6	
Cystatin-SN	P01037		-3.93		4.59	
BPI fold-containing family B member 2	Q8N4F0				-2.27	
Ig mu chain C region	P01871				-1.7	-2.36
Serotransferrin	P02787				-1.7	-2.36
Serpin B3	P29508			-2.29		
Immunoglobulin J chain	P01591				1.92	
14-3-3 protein sigma	P31947				2.01	
Zinc-alpha-2-glycoprotein	P25311				1.95	
Polymeric immunoglobulin receptor	P01833				-1.94	
BPI fold-containing family A member 2	Q96DR5				2.52	
Glyceraldehyde-3-phosphate dehydrogenase	P04406				-2.26	

Average ratios of statistically significant (p-value ≤ 0.05) up- and down-regulated sputum proteins are presented for the allergic rhinitis (AR), asthma and allergic rhinitis (asthma+AR) and non-allergic rhinitis (NAR) groups in comparison to the control group, and for AR and NAR groups in comparison to the asthma+AR group. More thorough identification parameters from the Mascot protein search can be found in supplement S1 Table.

### Clustering analyses reveal distinct patterns in protein regulation between asthmatics, allergic and nonallergic rhinitis patients and healthy controls

**Fig 3:**
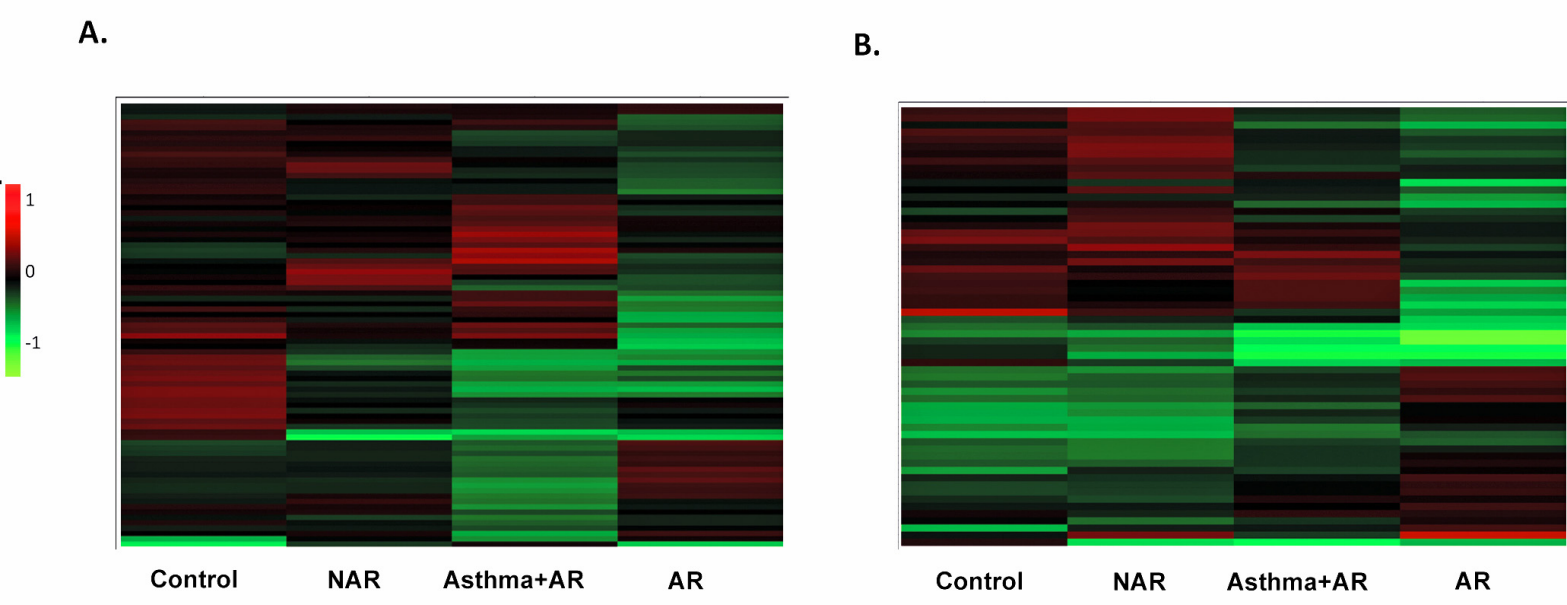
Heatmap of up- and down-regulated protein gel spots in sputum (A) and in nasal lavage fluid (B). Hierarchical clustering reveals the differences between the protein regulation of the groups, indicating dissimilarities between asthma (Asthma+AR), allergic rhinitis (AR) and healthy controls, whereas the nonallergic rhinitis (NAR) group shows the least differences to the up- or down-regulation of the healthy controls.

Hierarchical clustering revealed differences between the expression patterns of the four study groups in both sample types (Fig 3A and 3B). The most prominent difference was found between the control and AR groups, but the control and the asthma group also clearly differed from each other. Patterns of asthma+AR and AR groups in NLF samples are more similar to each other than the ones in sputum. The protein regulation in the NAR group most resembled that seen in the healthy controls (Fig 3). On the basis of the protein enrichment analysis, extracellular secreted proteins (65%, *p*-value 3.8E-9), especially saliva and plasma proteins, were abundant among the identified proteins from the IS samples. The outcome of the classification of proteins in biological processes pointed to one main Gene Ontology category, namely immune response (31%, *p*-value 1.1E-5).

The relationships between the significant abundance changes of the sputum proteins in the patient groups and those among the healthy controls are shown in Table 3. Decreased protein amounts were observed for most of the proteins in the AR and asthma groups, which shared five identifications. The NAR group showed the least changes in protein levels, with only one lower abundance protein. Table 3 contains also the corresponding relations found in the statistical analyses of the asthma and AR group and the other sets. The six proteins that emerged in the NAR group were all shared by the other groups. The amount of proinflammatory Protein S100-A8 (S10A8) is increased in asthma and AR group when compared to AR and control groups and decreased when compared to the protein levels in the NAR group.

Several significantly differentially up- or down-regulated proteins were identified in the 21 participant samples, such as Chitinase-3-like-protein 2 (CH3L2) also known as Human YKL39. Previously, Chitinase enzymes have been linked to asthma and allergic symptoms in human patient samples [24]. The function of Cysteine-rich secretory protein 3 (CRISP3), a protein highly expressed in salivary glands, neutrophils and eosinophils, is not yet fully understood, but has been postulated to have roles in innate immune defence and epithelial barrier function [25, 26]. Cystatin SN (CYTN), and Carbonic anhydrase 6 (CAH6) found from parotid gland and saliva also emerged in these analyses [27, 28]. Validation by Western blotting failed to support 2D-DIGE observations of the above mentioned proteins in the IS and NLF samples of the whole study population. This could be due to various proteoforms, the large dispersion of data values and variability of the saliva content in the samples. Commonly known saliva proteins such as lysozyme C (LYSC) and alpha-amylase 1 (AMY1) were also detected.

### Elevated Fatty acid binding protein 5 FABP5 levels among asthmatics

**Fig 4:**
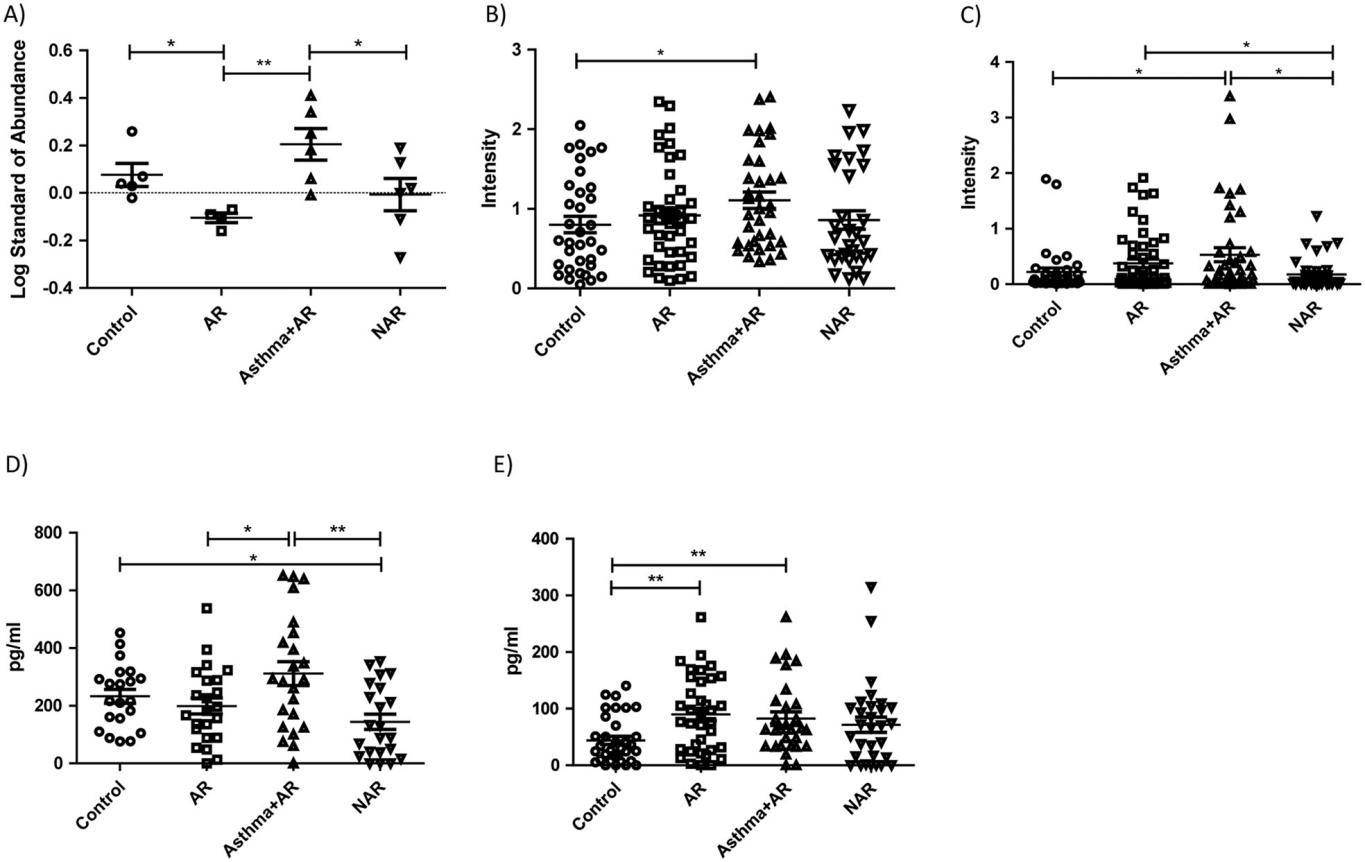
Immunological confirmation of findings. 2D-DIGE gel spot abundances for the FABP5 of the 21 induced sputum samples (A). Western blot analysis for 138 induced sputum samples (B) and for 158 nasal lavage fluid samples (C) with FABP5 antibody. ELISA results for vascular endothelial growth factor (VEGF) (D) and cysteinyl leukotriene (CysLT) (E) measurements from 90 nasal lavage fluid samples. The figures show mean with standard error of the mean. * p < 0.05, **p < 0.01. AR = allergic rhinitis, Asthma+AR = asthma and allergic rhinitis, NAR = nonallergic rhinitis.

Proteomic analysis of the 21 sputum samples revealed significant up-regulation of FABP5 gel spots in Asthma+AR group when compared to AR (Fig 4A). The FABP5 protein spot was not detected in the proteomic analysis of the NLF gels. As the identification score (S1 Table) was low for the faint IS gel spot, the concentration of FABP5 in NLF is most likely below the detection limit of the proteomic method utilised. Fatty acid proteins are a family of lipid chaperones consisting of nine different isoforms. They are involved in transporting lipids to specific locations in cell and thus playing a part in lipid metabolism, which is linked to inflammatory and metabolic processes [29]. FABP5, also known as epidermal FABP, is expressed in the epidermal cells of the tissues and also in the lungs, brain, heart, retina and spleen. FABP5 has also been linked to regulation of the inflammatory functions of macrophages and dendritic cells [30–32]. Since it is a transporter of fatty acids, FABP5 is suggested to play an important protective role against excessive oxidative damage to lipids during lung infection [33]. It has also been shown to promote Th17/Treg differentiation, pointing to a possible role in autoimmune and inflammatory diseases [34].

### Western blot validation of sputum and nasal lavage fluid confirms the up-regulation of FABP5 levels among asthmatic subjects

FABP5 showed the clearest regulation pattern changes in the antibody testing, and was thus chosen for validation in all sputum samples (Fig 4B, S1 Fig). The validation revealed the same abundance differences as the 2D-DIGE analysis with 21 samples (Fig 4A). The asthmatic group exhibited a statistically significant up-regulation of FABP5 in comparison to controls. The validation of FABP5 levels in all NFL samples revealed a similar pattern in the asthma and the control groups to that detected in the sputum analysis (Fig 4C). PCA with the immunoblot intensity values (S2A and S2B Fig) separates asthma+AR group clearly from other groups supporting the statistical analysis above. However, ROC analyses showed poor diagnostic performance of sputum and NLF FABP5 for predicting asthma (S3A and S3B Fig). We observed a positive correlation between the FABP5 protein intensity of the IS and NLF samples (r = 0.317, p<0.01) (S4A Fig).

### FABP5 levels correlate with vascular endothelial growth factor (VEGF) and cysteinyl leukotriene (CysLT) levels in nasal lavage fluid

FABP has been found to regulate VEGF induced airway inflammation [35]. Although VEGF was not identified from the protein gels, we detected a higher level of VEGF in NLF in asthmatic group than that in the AR and NAR groups (Fig 4D) with immunological method. The FABP5 abundance correlated positively with the IS and the VEGF NLF level (r = 0.371, p = 0.001), as did the FABP intensity in the NLF with that in VEGF NLF (r = 0.660, p<0.001) (S4B and S4C Fig). Since VEGF production can be activated by CysLTs [36], which have a well-established role in the pathogenesis of asthma [37], we also measured CysLT levels in NLF. The increased abundance of CysLT in nasal lavage correlated with the higher FABP5 levels in NLF (r = 0.535, p<0.001) and with the VEGF level in NLF (r = 0.611, p<0.001) (S4D and S4E Fig). Asthma+AR group also separates out in PCA with the obtained VEGF and CysLT concentrations (S2C Fig), which cluster together. As with FABP5 values above, ROC analyses showed poor diagnostic performance of CysLT and VEGF for predicting asthma in the applied study population (S3C and S3D Fig). Both VEGF and CysLT concentrations in sputum have been reported to be greater among asthmatic patients than non-asthmatic control subjects [38, 39]; thus they were not measured again in this study, as the DTT used in sputum processing might interfere with the antibody-based measurements by reducing the disulfide bonds of immunoglobulins.

## Discussion

Proteomic analysis of IS and NLF represent a noninvasive way to evaluate airway inflammation. The protein abundance patterns between the study groups were analysed with hierarchical clustering, a procedure in which proteins with similar expression profiles are clustered together. This comparison indicates that protein levels in AR and asthma groups differ from each other and from those of healthy controls, whereas the pattern in the NAR group resembles that of controls (Fig 3). We identified FABP5 in the sputum proteome assessment and noted that it was up-regulated in both the sputum and NLF samples of allergic asthmatics with AR supporting the hypothesis that there are common underlying inflammatory processes in the upper and lower airways.

The asthmatic subjects had normal mean spirometric and exhaled nitric oxide values and most of them were not taking any regular asthma medication, indicating that they mainly had mild asthma. The severity of nasal symptoms in all rhinitis groups was also low [16, 40]. Therefore, the findings of this study may be generalized to subjects with current mild allergic asthma, AR and NAR rather than those with more severe diseases. The proteome analysis was performed in nonsmokers, the level of current smoking was low in the study population and the groups did not differ significantly in terms of smoking. Thus we presume that smoking did not have any significant effect on our results.

Although asthma and AR are both characterised by airway inflammation [3], present methods for monitoring inflammation are of limited value in clinical practice. IS is widely used to assess airway inflammation in asthma, but there are some limitations associated with this procedure. One of the major practical limitations is the failure to obtain suitable sputum samples from a substantial proportion of patients [41]. In addition, DTT is commonly used to separate IS fluid phase from the cells, possibly affecting the analyses of the cells and fluid phase biomarkers [42]. In contrast, NLF can be obtained from almost all subjects and cells can be separated without chemical treatment. The eosinophil count in NLF has been shown to be a good predictor of sputum eosinophilia among asthmatic subjects [43]. Most of the proteins in the NLF and in the IS were identical, and the identified proteins overlap with the previously described proteins detected in bronchoalveolar fluid and IS. These findings provide further confirmation of the usefulness of NLF in upper and lower airways epithelia research, and suggest that NLF might be used as a surrogate to examine lower airway inflammation among asthmatic subjects.

The underlying mechanisms linking allergenic reactions to impaired lung function in the development of the asthma phenotype are unclear, but they may be related to factors derived from the airway epithelium [44]. The airway epithelium is much more than a passive barrier to the external environment [45]: it is also actively involved in chronic disease processes such as airway remodeling. Besides being structural components of cell membrane and an energy source, lipids are important signaling molecules modulating cellular functions. FABPs are small abundantly expressed lipid-binding proteins, with a molecular weight of approximately 15 kDa, which play an important role in the regulation of glucose and lipid homeostasis as well as in inflammation via their actions on adipocytes and immune cells [30, 46]. These lipid chaperones are mainly found in cytoplasm, but they can be translocated to nucleus after ligand binding [47, 48]. FABP5 has also been identified from the *in vitro* secretome of macrophages [49], but its true secretion to body fluids needs to be verified.

All nine tissue-specific FABPs bind long-chain fatty acids, but there are differences in ligand selectivity, binding affinity and binding mechanism as a result of structural differences in the ligand binding pocket between isoforms [50]. It seems that the needs of target cells determine the affinity and even selectivity of the major isoform present at different sites [47]. Of the studied fatty acids, linoleic and arachidonic acids are of particular interest as they can be metabolized into bioactive lipid mediators, eicosanoids, which may function as pro- and anti-inflammatory mediators. Both of these fatty acids have been shown to be activators of FABP5 [48]. FABP1, although mainly produced by liver, was related to the development of aspirin induced bronchoconstriction in asthmatics [51]. FABP5 has earlier been linked to Th17/Treg differentiation, oxidative damage and inflammatory responses [30–32, 34]. FABPs, including FABP5, take also part in signaling pathways by transactivating nuclear transcription factors like peroxisome proliferator-activated receptors (PPARs) [47,48], which link metabolic changes to genome expression and are involved in chronic inflammatory disease [52]. PPAR-FABP5 pair is also suggested to have a role in lung surfactant synthesis in alveolar type II cells [53]. Previous studies have suggested that FABP4 protein plays a significant role in allergic asthma, although the exact mechanism underlying this effect is not clear [54]. FABP4 has been identified as a target of the VEGF/VEGFR2 signaling pathway [35] and FABP5 has been shown to up-regulate the expression of its possible downstream mediator, VEGF [55]. FABP-deficient mouse model studies have shown that FABP4 and FABP5 are able to compensate for the lack of one of these two proteins [30], thus the reported regulations between FABP4/FABP5 and VEGF may apply to both of them. VEGF is an epithelium-derived cytokine with well-defined involvement in angiogenesis, fibrosis, and smooth muscle hypertrophy and hyperplasia in the asthmatic airway as well as in Th2 type inflammation [56, 57]. The molecular components involved in VEGF associated airway remodeling and inflammation in asthma have not been well characterized. Our findings that the increased FABP5 levels correlate with the VEGF levels in the airway samples of asthmatic subjects supports the proposal of some kind of linkage between FABPs and VEGF.

The cytokine production of immune cells is modulated by eicosanoids such as leukotrienes and prostaglandins, which are derivatives of long-chain fatty acids. The transport and storage of these fatty acids requires an interaction with carrier proteins such as FABPs. CysLT subgroup members are released by eosinophils, macrophages and mast cells in the airways and they have several important pathogenic roles in asthma, such as promoting inflammation. They might also contribute to maintaining persistent inflammation. The role of CysLTs in airway remodeling could be mediated through VEGF, as CysLTs have been shown to up-regulate VEGF production [36]. Our results suggest that FABP5 may be a modulator between CysLTs and VEGF-mediated cell signaling cascades. The evidence we obtained of the role of FABP5 in asthmatic inflammation in airways needs to be confirmed in further studies on more severe asthma patients and larger groups.

## Conclusions

The overall goal of the study was to investigate potential mechanisms by which a chronic allergic reaction could lead to the induction of airway epithelium factors that might influence lung function and the pathogenesis of asthma. Our findings indicate that FABP5 may contribute to the airway remodeling and inflammation in asthma by inducing VEGF production. Taken together, we propose that pro-inflammatory conditions alter fatty acid signaling via multiple mechanisms that might impact the balance of lipid metabolism and alter the course of inflammatory diseases such as asthma. Further studies are needed to confirm our suggestive results and to clarify the existence of the mechanisms and the additional molecular components contributing to it.

## Supporting Information

S1 Fig Western blot image of FABP5 validation from 23 sputum (A) and 24 NLF samples (B). Starting from the left, a molecular weight marker (Precision Plus Protein, Dual color, Bio-Rad Laboratories) followed by the pooled sample and five to six patient samples per each group are represented as an example.
(PDF)

S2 Fig Principal component analysis with the validation results for FABP5, CysLT and VEGF. Euclidean distances were compared between the study groups. FABP5 in IS (A), FABP5 in NLF (B), CysLT and VEGF in NLF (C). AR = allergic rhinitis, Asthma.AR = asthma and allergic rhinitis, NAR = nonallergic rhinitis, Contr = healthy controls.
(PDF)

S3 Fig Receiver operating characteristics curve (ROC) analyses of the sensitivity and the specificity of FABP5 for the diagnosis of asthma. Asthma group was compared to all the other groups. A) ROC FABP5 in sputum as well as B) FABP5 C) VEGF and D) CysLT in nasal lavage fluid for predict asthma. AUC = area under the curve.
(TIF)

S4 Fig Correlations between FABP5 intensities and VEGF and CysLT levels. Correlations between FABP5 Western Blot band intensity in induced sputum (IS) and in nasal lavage fluid (NLF) (A), FABP5 intensity in IS and VEGF level in NLF (B), FABP5 intensity in NLF and VEGF level in NLF (C), FABP5 intensity in NLF and CysLT level in NLF (D) and VEGF and CysLT levels in NLF (E).
(TIF)

S1 Table List of identified proteins from 2D-DIGE analysis of sputum samples. Proteins from sputum samples were identified from the picked gel spots by tandem mass spectrometry and Mascot software. Student's t-test and the Average (Av.) Ratio (with significance levels of < 0.05 and Av. Ratio ≥ |1.5|, respectively) for comparing allergic rhinitis (AR), asthma with allergic rhinitis (asthma + AR), non-allergic rhinitis (NAR) and healthy controls to one another were obtained from DeCyder software.
(XLSX)
