# Supplementary material for: Level of Fatty Acid Binding Protein 5 (FABP5) Is Increased in Sputum of Allergic Asthmatics and Links to Airway Remodeling and Inflammation
Source: PLoS One. 2015 May 28;10(5):e0127003. doi: 10.1371/journal.pone.0127003 (PMC4447257; doi:10.1371/journal.pone.0127003)
Supplement: S1 Fig — Starting from the left, a molecular weight marker (Precision Plus Protein, Dual color, Bio-Rad Laboratories) followed by the pooled sample and five to six patient samples per each group are represented as an example. (PDF) [file pone.0127003.s001.pdf]

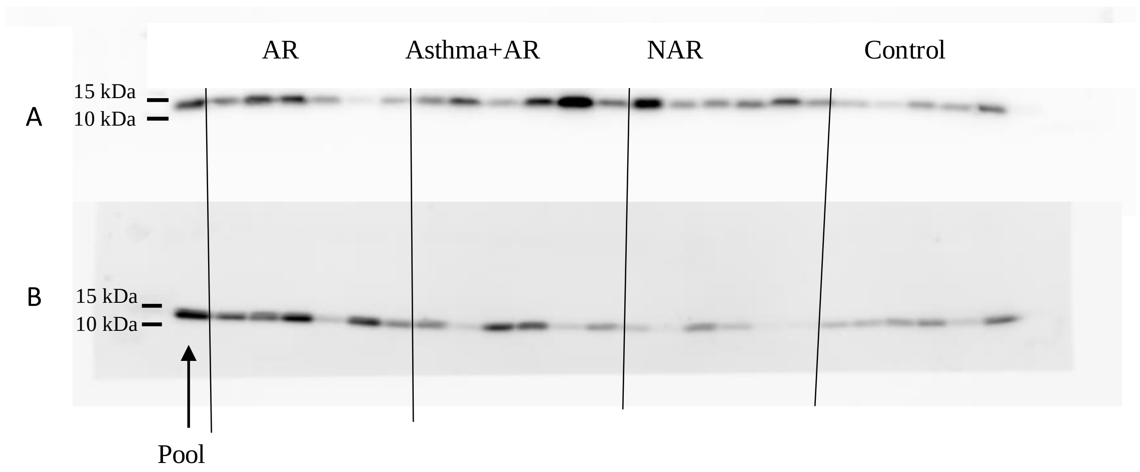

S1 Fig. Western blot image of FABP5 validation from 23 sputum (A) and 24 NLF samples (B). Starting from the left, a molecular weight marker (Precision Plus Protein, Dual color, Bio-Rad Laboratories) followed by the pooled sample and five to six patient samples per each group are represented as an example.
