# Supplementary material for: Level of Fatty Acid Binding Protein 5 (FABP5) Is Increased in Sputum of Allergic Asthmatics and Links to Airway Remodeling and Inflammation
Source: PLoS One. 2015 May 28;10(5):e0127003. doi: 10.1371/journal.pone.0127003 (PMC4447257; doi:10.1371/journal.pone.0127003)
Supplement: S2 Fig — Euclidean distances were compared between the study groups. FABP5 in IS (A), FABP5 in NLF (B), CysLT and VEGF in NLF (C). AR = allergic rhinitis, Asthma.AR = asthma and allergic rhinitis, NAR = nonallergic rhinitis, Contr = healthy controls. (PDF) [file pone.0127003.s002.pdf]

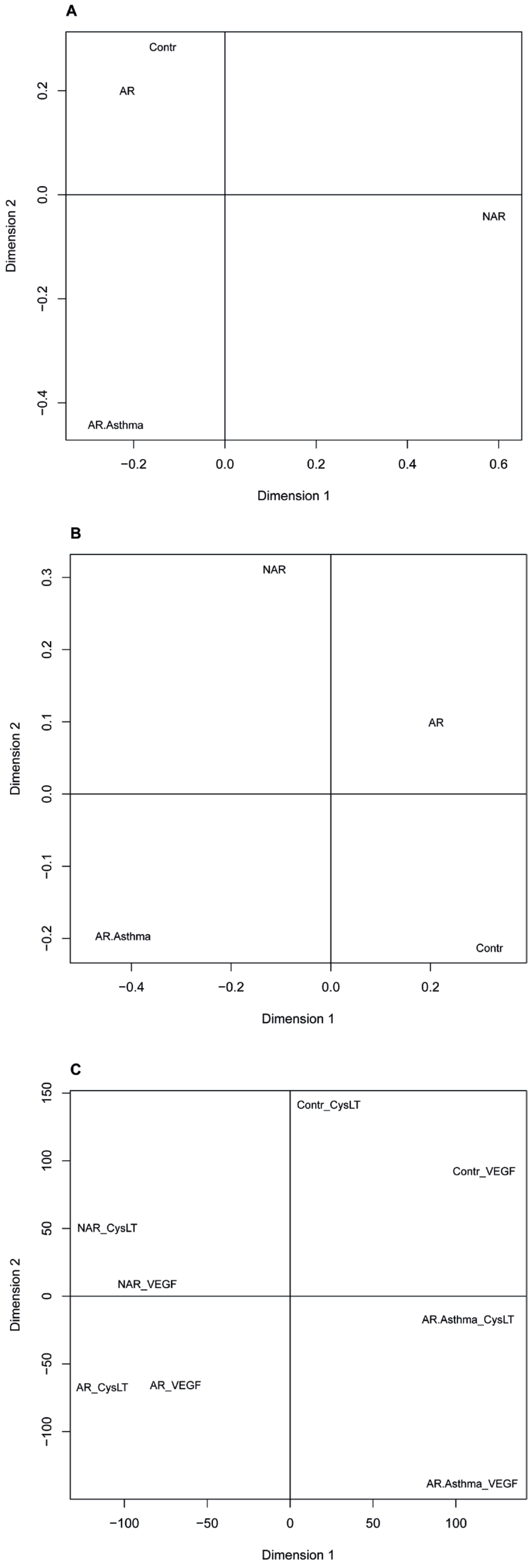

S2 Fig. Principal component analysis with the validation results for FABP5, CysLT and VEGF. Euclidean distances were compared between the study groups. FABP5 in IS (A), FABP5 in NLF (B), CysLT and VEGF in NLF (C)**.** AR= allergic rhinitis, Asthma.AR= asthma and allergic rhinitis, NAR= nonallergic rhinitis, Contr= healthy controls.
